# Preventive Effects of β-Cryptoxanthin, a Potent Antioxidant and Provitamin A Carotenoid, on Lifestyle-Related Diseases—A Central Focus on Its Effects on Non-Alcoholic Fatty Liver Disease (NAFLD)

**DOI:** 10.3390/antiox11010043

**Published:** 2021-12-26

**Authors:** Azusa Nishino, Takashi Maoka, Hiroyuki Yasui

**Affiliations:** 1 Applied Research Laboratory, Ezaki Glico Co., Ltd., 4-6-5 Utajima, Nishiyodogawa-ku, Osaka 555-8502, Japan; azusa.nishino@glico.com; 2 Research Institute for Production Development, 15 Morimoto-cho, Shimogamo, Sakyo-ku, Kyoto 606-0805, Japan; maoka@mbox.kyoto-inet.or.jp; 3 Department of Analytical and Bioinorganic Chemistry, Division of Analytical and Physical Sciences, Kyoto Pharmaceutical University, 5 Nakauchi-cho, Misasagi, Yamashina-ku, Kyoto 607-841, Japan

**Keywords:** β-cryptoxanthin, potent antioxidant, provitamin A, retinoids, BCMO1, NAFLD, lifestyle-related diseases, oxidative stress, insulin resistance, PPARγ

## Abstract

Humans usually get dietary carotenoids from foods such as green and yellow vegetables and algae. Carotenoids have been reported to effectively reduce the risk of developing lifestyle-related diseases. β-Cryptoxanthin, which is an antioxidative carotenoid and a type of provitamin A, is metabolically converted to vitamin A. β-Cryptoxanthin has recently gained attention for its risk-reducing effects on lifestyle-related diseases, especially on non-alcoholic fatty liver disease (NAFLD), from epidemiological, interventional, and mechanistic studies. Retinoids (vitamin A) have also been reported to be useful as a therapeutic agent for NAFLD. Provitamin A is known to serve as a supply source of retinoids through metabolic conversion by the regulated activity of β-carotene 15,15′-monooxygenase 1 (BCMO1) to the retina only when retinoids are deficient. From mechanistic studies using NAFLD-model mice, β-cryptoxanthin has been shown to contribute to the improvement of NAFLD through a multifaceted approach, including improved insulin resistance, suppression of oxidative stress and inflammation, a reduction of macrophages and a shift of their subsets, and control of lipid metabolism by peroxisome proliferator-activated receptor (PPAR) family activation, which are also expected to have clinical applications. β-Cryptoxanthin has the potential to prevent lifestyle-related diseases from different angles, not only as an antioxidant but also as a retinoid precursor.

## 1. Introduction

Carotenoids, a representative phytochemical, is a generic term for a group of compounds with the underlying structure of a 40-carbon polyene skeleton biosynthesized in green and yellow vegetables and algae and characterized by high levels of anti-oxidative activities [1,2,3,4]. The SOAC (singlet oxygen absorption capacity) method to assess the total quenching activity of singlet oxygen (^1^O_2_) by carotenoids included in foods and plants has been established, where the quenching rates of ^1^O_2_ by eight carotenoids and α-tocopherol were measured by using the competition reaction method. As a summary of the correctly measured SOAC values for carotenoids, the SOAC values decrease in the following order: lycopene > astaxanthin > β-carotene ≈ capsanthin ≈ zeaxanthin ≈ α-carotene > lutein > β-cryptoxanthin >> α-tocopherol. However, the actual difference among the SOAC values of carotenoids is not remarkable. The value of lycopene is only 1.8 times larger than that of β-cryptoxanthin, while the SOAC value of lycopene is 123 times larger than that of α-tocopherol [5].

**Figure 1:**
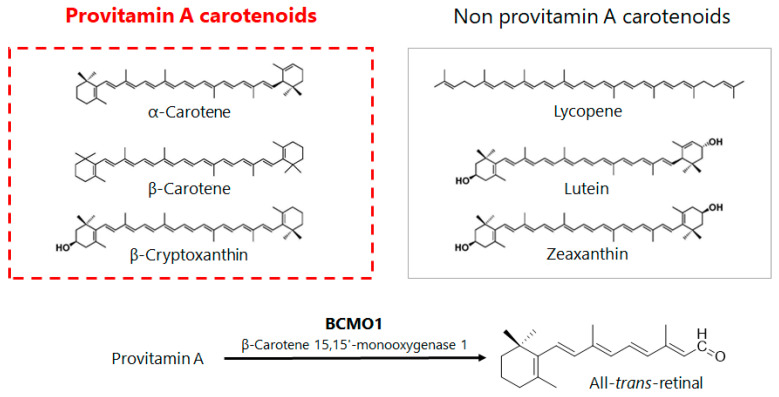
Chemical structure of dietary provitamin A and non-provitamin A carotenoids normally present in human blood, and metabolic conversion of provitamin A to all-trans-retinal by β-carotene 15,15′-monooxygenase 1 (BCMO-1): Carotenoids are defined as yellow to red natural colorants bio-synthesized by plants and algae, characterized to express high anti-oxidative activities, and found to have physiologically defensive functions on visual performance, life-style related diseases and cancers. Six kinds of dietary carotenoids are normally present in human blood.

As humans consume carotenoids in their diets on a daily basis, six types of dietary carotenoids are typically found in human blood: Lycopene, α-carotene, β-carotene, β-cryptoxanthin, lutein, and zeaxanthin [6,7]. Of these, α-carotene, β-carotene, and β-cryptoxanthin are metabolically converted to vitamin A in vivo and are therefore also called provitamin A (Figure 1) [8]. Various epidemiological and intervention studies have been conducted on these six dietary carotenoids, and their diverse physiological functions have been verified in research on their preventive effects against cancers and other lifestyle-related diseases [9,10,11,12]. The types of carotenoids attracting attention are changing in response to shifts in the historical background and social environment. Lutein, for example, has garnered attention for its physiological functions in visual performance because of recently increased macular degeneration and other visual impairments. Lutein has been reported to be distributed in the retina and macula [13,14], and is thus believed to protect the eye from light damage. Since age-related macular degeneration occurs more frequently in Europe and the United States, there is a strong interest in lutein and research has been mainly developed on its effects on visual performance in these regions.

At the same time, lifestyle-related diseases are rapidly increasing, which has been a serious issue facing modern society. Carotenoids and carotenoid-rich green and yellow vegetables have been reported to effectively reduce the risk of developing lifestyle-related diseases [15,16]. In particular, β-cryptoxanthin has recently gained attention for its risk-reducing effects on lifestyle-related diseases. Since β-cryptoxanthin has a substitution of a hydroxyl group in the C3 position of β-carotene, its end structure with the hydroxyl group is polar and the other end structure composed of a hydrocarbon is non-polar. That is, β-cryptoxanthin has the polar group in only one side of its whole molecule. Additionally, β-cryptoxanthin is one of the few xanthophylls with provitamin A activity because of producing retinal by cleavage in the middle of the molecule [17]. β-Cryptoxanthin is widely distributed in nature in fruits and vegetables such as red pepper, persimmons, and loquat and is particularly abundant in the Satsuma mandarin (*Citrus unshu Marc*.), a citrus species indigenous to Japan. Japanese people who habitually consume Satsuma mandarin oranges are reported to have higher blood levels of β-cryptoxanthin than people in other countries [18]. The intake frequency of Satsuma mandarin oranges reportedly correlates with the concentration of β-cryptoxanthin in the blood [19]. Given this background, β-cryptoxanthin has been actively studied in Japan for its physiological functions. This review describes the effects of β-cryptoxanthin on lifestyle-related diseases, especially on non-alcoholic fatty liver disease (NAFLD), from epidemiological, interventional, and mechanistic studies.

## 2. Relationship between the Intake of β-Cryptoxanthin and Risk of Lifestyle-Related Diseases in Terms of Epidemiological Studies

Among the representative epidemiological studies on β-cryptoxanthin is the “Mikkabi Cohort Study” conducted in Japan (Table 1). The epidemiological study of residents in Mikkabi Town, Shizuoka Prefecture, a major producer of Satsuma mandarin oranges, has continuously investigated the correlation between serum concentrations of six dietary carotenoids (Figure 1) and the risk of developing lifestyle-related diseases since 2005.

Based on the carotenoid serum concentrations at baseline, the subjects were classified into three groups of high, medium, and low concentration, and then the risk of developing lifestyle-related diseases was compared among the groups and analyzed on the basis of follow-up surveys conducted over the next ten years. Metabolic syndrome (MS) was diagnosed on the basis of a BMI of 25 or higher and two or more of the following specified items: hyperglycemia, hypertension, and dyslipidemia. β-Carotene concentrations showed the most significant inverse correlation when the risk of developing MS was analyzed; α-carotene and β-cryptoxanthin also showed similar tendencies, although a significant difference was not observed. Regarding the risk of developing dyslipidemia, one of the diagnostic criteria for MS, a significant inverse correlation was shown with the concentrations of α-carotene, β-carotene, and β-cryptoxanthin, or provitamin A carotenoids [20].

While the risk of developing type 2 diabetes was analyzed using fasting blood glucose of 7 mmol/L or higher as a diagnostic criterion, the groups with higher α-carotene and β-cryptoxanthin concentrations were found to have a significantly lower risk than the lower concentration group [21]. An epidemiological study in Finland also reported that β-cryptoxanthin intake reduces the risk of developing type 2 diabetes [22], which is consistent with the results of the Mikkabi Cohort Study (Table 1). At the same time, a follow-up study on the risk of developing non-alcoholic liver dysfunction using serum alanine aminotransferase (ALT) concentration, a marker of liver damage, also revealed that the risk of developing NAFLD was significantly reduced in the high serum provitamin A concentration group [23]. Furthermore, an epidemiological study conducted in China on serum carotenoid concentrations and NAFLD described a significant inverse correlation between serum carotenoid concentration and NAFLD progression diagnosed by abdominal ultrasonography [24]. This correlation strength was greater for provitamin A than for non-provitamin A, which is consistent with the results from the Mikkabi study (Table 1). The cross-sectional and interventional studies of Japanese patients with NAFLD found that serum levels of β-cryptoxanthin were significantly lower in patients with NAFLD than in healthy subjects and that oral administration of β-cryptoxanthin to patients with NAFLD significantly reduced the liver damage markers ALT, aspartate amino group transferase (AST) and γ-glutamyl transpeptidase (γ-GTP), the inflammatory marker interleukin (IL)-6, and the oxidative stress marker oxidized LDL compared to placebo [25]. These results indicate that oral intake of β-cryptoxanthin is effective not only in reducing the risk of developing NAFLD but also in improving the post-disease conditions and that the reduction of oxidative stress is one of the mechanisms involved.

Oxidative stress has been found to be deeply involved in obesity, diabetes, and NAFLD, and the high antioxidant activity of carotenoids is thought to contribute to preventing and improving these diseases. Taking fully into account the epidemiological studies mentioned above in totality, however, significant correlations with reduced risk of developing lifestyle-related diseases to high serum concentrations of mainly provitamin A, α-carotene, β-carotene, and β-cryptoxanthin are rather limited, and no significant correlations have been found with non-provitamin A, lutein, zeaxanthin, and lycopene in many cases. Since the antioxidant activity of carotenoids is generally higher in non-provitamin A than in provitamin A, it can be inferred that the involvement of carotenoids in lifestyle-related diseases does not depend solely on their antioxidant activity and instead there are other mechanisms of action, such as supplementation of provitamin A converted to vitamin A (retinoids) in the body.

## 3. Retinoid Metabolic Disorders in Non-Alcoholic Fatty Liver Disease (NAFLD)

**Figure 2:**
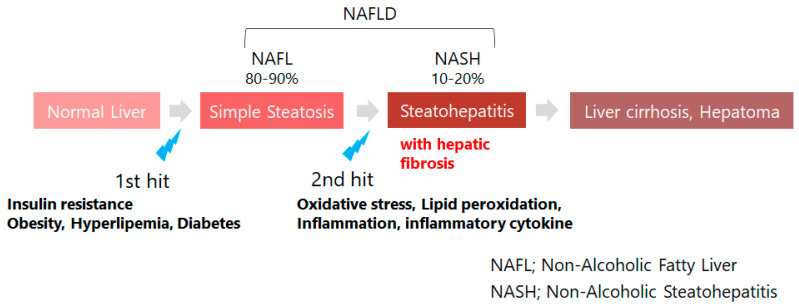
Non-alcoholic fatty liver disease (NAFLD) and its inducing factors: NAFLD is recognized as the hepatic phenotype of metabolic syndrome, which is closely related with lifestyle diseases such as obesity, type 2 diabetes, and disorder of lipid metabolism. The issue of concern is the acute increase in patients with obesity in the world. Metabolic disorder of retinoids (vitamin A) is observed in patients with impaired hepatic function including NAFLD, where almost 80% of retinoids are stored in hepatic stellate cells of human.

NAFLD is closely associated with obesity, diabetes, dyslipidemia, and other lifestyle-related diseases and is also referred to as the hepatic phenotype of MS. The rapid increase in the number of patients has become a social problem, where 25% of the global adult population is reported to be affected with NAFLD [26,27]. Nevertheless, no solid treatment has been established to date. The 80 to 90% of patients with NAFLD have simple fatty liver (NAFL) with no progression, and 10 to 20% progress to non-alcoholic steatohepatitis (NASH) with liver fibrosis, which becomes severe, leading to cirrhosis and liver cancer (Figure 2). The “two-hit theory” has been widely recognized, in which insulin resistance and oxidative stress are deeply involved in the pathogenesis of NAFLD [28,29], and serum provitamin A concentration is inversely correlated with these factors and the risk of developing NAFLD (Section 2). Provitamin A is a source of vitamin A (retinoid) through metabolic conversion in vivo, while abnormalities in retinoid metabolism in vivo are known to contribute to NAFLD and other liver diseases [26]. Here we would like to discuss the relationship between abnormal retinoid metabolism and provitamin A.

**Figure 3:**
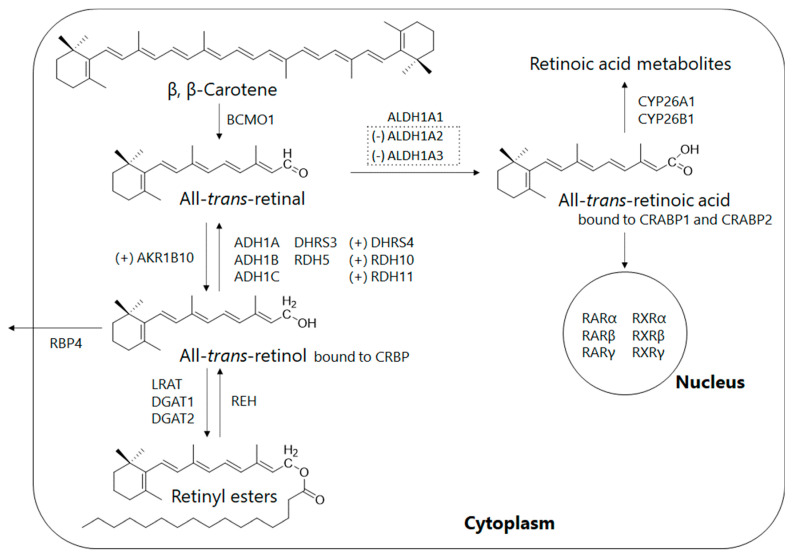
Intracellular metabolic pathway of retinoid in the liver: The all-trans-retinol binds to cellular retinol-binding protein (CRBP) and is esterized by lecithin retinol acyltransferase (LRAT) and diacylglycerol acyltransferases (DGAT1, DGAT2) for intracellular storage, whereas retinol bound with retinol-binding protein 4 (RBP4) is transported into other tissues via blood circulation. Retinol is oxidized into all-trans-retinoic acid in the liver via two stages as follows: The reversible oxidation/reduction between retinol and retinal is regulated by three kinds of enzymes: (i) retinal dehydrogenases (RDH5, RDH10, RDH11), (ii) alcohol dehydrogenases (ADH1A, ADH1B, ADH1C); and (iii) membrane-bound short-chain dehydrogenases (DHRS3, DHRS4). The irreversible oxidation of retinal to all-trans-retinoic acid is maintained by retinaldehyde dehydrogenases 1 (ALDH1A1, ALDH1A2 and ALDH1A3). All-trans-retinoic acid, which binds to cellular retinoic acid-binding proteins (CRABP1 and CRABP2), is eliminated from the body by the cytochrome P450 (CYP26A1 and CYP26B1)-dependent oxidation. After entering the nucleus, all-trans-retinoic acid binds to a retinoic acid receptor/retinoic X receptor (RAR/RXR) heterodimer and stimulates transcription of target genes. In NAFL and NASH, the (+) symbol means an increased expression of aldo-keto reductase 1 B10 (AKR1B10), whereas the (−) symbol means a reduced expression of retinaldehyde dehydrogenase 1. Provitamin A is metabolized to all-trans-retinal by β-carotene 15,15′-monooxygenase 1 (BCMO-1) in vitamin A deficient states such as NAFL and NASH.

**Figure 4:**
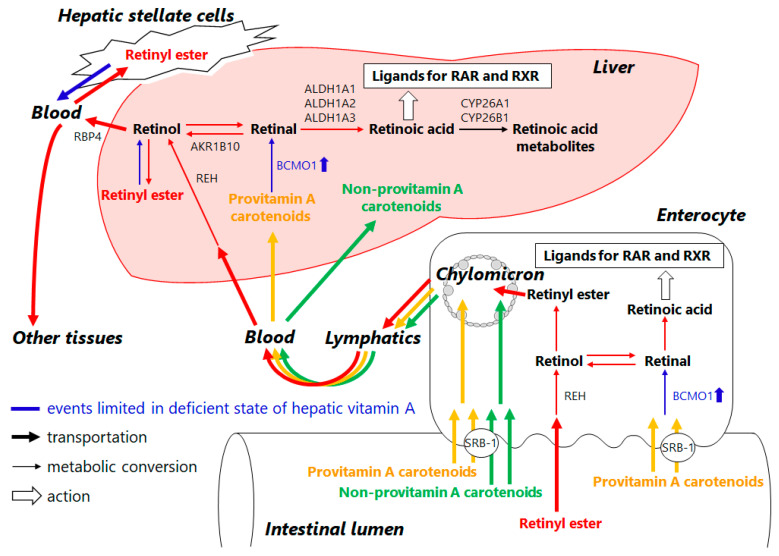
Transportation and metabolism of retinoids in the normal and vitamin A deficient states of the small intestine and liver: Retinyl ester is usually provided by the daily diet and transported via chylomicrons into the liver. In the normal state of the liver, retinoids are sufficient and stored as retinyl ester mainly in the hepatic stellate cells, while, in the vitamin A deficient state of the liver (shown by blue color symbols and events), dietary retinoids are insufficient and retinyl ester stored in the hepatic stellate cells are consumed throughout the body. Additionally, upregulation of BCMO-1 metabolically converts provitamin A carotenoids to retinal for complement of retinoids. REH: retinyl ester hydrolase, RBP4: retinol-binding protein 4, SRB-1: scavenger receptor class B type 1, BCMO-1: β-carotene 15,15′-monooxygenase 1.

Retinoids are essential micronutrients in humans and are converted into retinol, retinyl esters, retinal, and retinoic acid by enzymatic metabolism in vivo to perform various functions (Figure 3). Retinal, for example, is present as a cofactor in rhodopsin, a photoreceptor molecule in the retina, and is vital for sustaining visual performance [29]. Retinoic acid, which is formed from retinal by oxidation, plays important roles throughout the body as a ligand for the nuclear receptors such as retinoic acid receptor (RAR) and retinoid X receptor (RXR), including cell proliferation and differentiation, maintenance of homeostasis, and regulation of the immune system (Figure 3). Despite being essential for the maintenance of biological functions, retinoids cannot be synthesized in the human body, and therefore humans depend on their diet for supply: retinoids are consumed as retinyl esters from animal foods and as provitamin A from plant foods (Figure 4). Developed countries have ample amounts of retinoids in their diets, while in some regions in developing countries, health problems due to inadequate retinoid intake are still an issue [30].

Both dietary retinyl esters and provitamin A are absorbed from small intestinal epithelial cells and then taken up in chylomicrons for release into the lymphatic system [31]. When retinoids are deficient in the body, dietary provitamin A is converted to retinal by β-carotene 15,15′-monooxygenase 1 (BCMO1), which is highly expressed in the small intestine. BCMO1 is also expressed in the liver, where it is thought to undergo the same metabolic conversion as in the small intestine (Figure 3 and Figure 4) [32]. The liver is a retinoid-storing tissue, and hepatic stellate cells in particular store approximately 70% of the total retinoids in the body as retinyl esters and supply them to the whole body as needed (Figure 4) [33]. When hepatic stellate cells are activated due to hepatic injury, stored retinoids are lost as fat droplets, resulting in progressive hepatic fibrosis (Figure 4) [34]. In other words, abnormalities in retinoid metabolism resulting from liver injury may be responsible for retinoid depletion even when dietary intake is sufficient.

Pettinelli et al. analyzed the gene expression levels of enzymes associated with retinoid metabolism in the livers of healthy subjects and patients with NAFLD [35]. The results suggest that gene expression of aldehyde dehydrogenases, retinaldehyde dehydrogenase family 1, members A2 and A3 (ALDH1A2, ALDH1A3), which produce retinoic acid through retinal oxidative metabolism, are suppressed in patients with NAFLD (NAFL and NASH), compared to healthy subjects, resulting in decreased retinoic acid production in the body. In addition, gene expression of aldo-keto reductase family 1 member B10 (AKR1B10), which catalyzes the reaction of retinal to retinol, was elevated in NASH patients compared to healthy subjects and NAFL patients, suggesting that retinal, the substrate of retinoic acid, is decreased (Figure 3 and Figure 4) [35]. At the same time, serum retinol levels were significantly higher in NAFLD patients than in healthy subjects. Another study also reported that the hepatic retinol stores in NASH patients were lower than those in NAFL patients [36]. These in turn imply that in NAFLD, in addition to the release of stored retinoids due to activation of hepatic stellate cells, abnormal retinoid metabolism enhances retinol production and release into the blood, resulting in depletion of retinoids in the liver and decreased bioavailability in the body (Figure 4). AKR1B10, which is overexpressed in NASH, is known to also be overexpressed in cells of several cancer types, including hepatocellular carcinoma and lung cancer. AKR1B10 inhibitors are also targets for drug discovery research in cancer treatments, as AKR1B10 disrupts RAR- and RXR-mediated biological control and is responsible for increased proliferation of cancer cells [37].

Based on these findings and results, retinoic acid depletion resulting from retinoid deficiency is thought to cause abnormalities in RAR- and RXR-mediated signaling, disrupt biological homeostasis, and be responsible for various diseases. Retinoids have been reported to be useful as a therapeutic agent for NAFLD [38], but on the other hand, overconsumption of them can cause adverse effects, and caution should be taken: a continuous high intake is therefore not recommended for the purpose of disease prevention [39]. Provitamin A, in contrast, is free of excess symptoms and is known to serve as a supply source of retinoids through metabolic conversion by the regulated activity of BCMO1 to the retinal only when retinoids are deficient (Figure 4) [40]. Thus, leakage of stored retinoids due to abnormal retinoid metabolism or activation of hepatic stellate cells results in retinoid deficiency, in which metabolic conversion or consumption of provitamin A is expected to be enhanced. Concomitantly, the concentration of provitamin A in tissues is estimated to decrease. This mechanism may be responsible for the inverse correlation between the incidence and progression of NAFLD and serum provitamin A concentration, as observed in the epidemiological study in Section 2. Furthermore, β-cryptoxanthin was also found to act independently as a ligand for RARs [41]. Unlike retinoids, provitamin A has almost no risk of overdose due to continuous intake. Maintaining high levels of provitamin A in the body is expected to be useful in preventing NAFLD and other diseases.

## 4. Mechanisms of Action of β-Cryptoxanthin for Treatment of NAFLD

In Section 3, we discussed the relationship between NAFLD and serum provitamin A concentrations observed in epidemiological studies from the perspective of retinoid metabolism. Here, we will focus on the mechanism by which β-cryptoxanthin inhibits the onset of NAFLD, as revealed in animal studies. Although the mechanism of NAFLD pathogenesis remains unclear, the most widely accepted hypothesis is the “two-hit theory.” In the first hit, fat accumulation in the liver due to insulin resistance caused by lifestyle-related diseases leads to the NAFL states. Under this condition, oxidative stress-induced lipid peroxidation, activation of inflammatory cytokines and other conditions are added in the second hit, which promotes the progression to NASH accompanied by liver fibrosis (Figure 2). Based on this hypothesis, insulin resistance and oxidative stress are considered the two major factors in the pathogenesis of NAFLD, as mentioned in Section 3.

**Figure 5:**
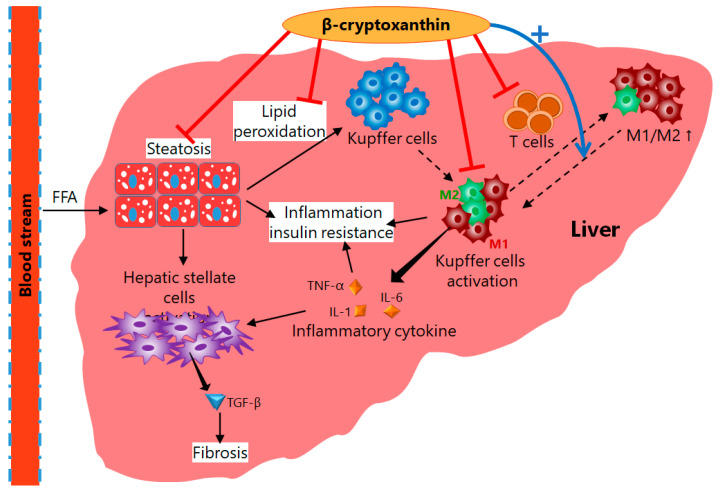
Control mechanism of dietary β-cryptoxanthin against progression of NAFLD: β-Cryptoxanthin inhibits not only lipid accumulation but also acts as an antioxidant and suppresses lipid peroxidation and inflammation, and thus results in improved insulin resistance in the liver. Additionally, β-cryptoxanthin prevents Kupffer cell activation by changing the M1/M2 status to M2 predominant. Consequently, β-cryptoxanthin controls progression of NAFLD by various biological and defensive activities.

In general, NASH model mice are produced by feeding a high-cholesterol and high-fat diet to normal mice. However, mice fed a NASH-inducing diet and β-cryptoxanthin simultaneously suppressed fat accumulation and fibrosis in the liver, and the induction of NASH was inhibited [42,43]. In addition, β-cryptoxanthin also improved insulin resistance in the NASH model mice. At the same time, the phosphorylation reaction in the insulin signaling pathway, which was attenuated in the NASH model mice, was activated (restored) by β-cryptoxanthin intake, suggesting that β-cryptoxanthin improves insulin sensitivity [43]. This indicates that β-cryptoxanthin prevents fat accumulation in the liver and blocks NAFL by improving insulin resistance in the “first hit” (Figure 5).

Liver fibrosis, which is observed in the progression from NAFL to NASH, is caused from the activation of hepatic stellate cells with tumor necrosis factor-α (TNF-α), IL-1, and IL-6 produced by Kupffer cells, hepatic macrophages. Gene expression of these pro-inflammatory cytokines was upregulated in the NASH model mice, but the administered β-cryptoxanthin suppressed the upregulation of these genes (Figure 5), which were closely associated with attenuation of the phosphorylation of JNK, p38 MAPK, and NF-ĸB p65 [43]. Moreover, the administration of β-cryptoxanthin decreased the production and accumulation of lipid peroxides in the liver, indicating that oxidative stress was also reduced [42,43]. These results suggest that β-cryptoxanthin suppresses lipid peroxidation by reducing oxidative stress, a “second hit” factor, and also prevents activation of hepatic stellate cells by suppressing the gene expression of inflammatory factors induced under oxidative stress, thus inhibiting the progression from NAFL to NASH (Figure 5).

A noteworthy physiological function of β-cryptoxanthin is to alter a subset of macrophages. There are two subsets of macrophages: the M1 type, which promotes inflammation and oxidative stress, and the M2 type, which acts as an anti-inflammatory and tissue repair agent. In obesity, the M1/M2 macrophage polarization in adipose tissue is known to shift toward M1 dominance and induces chronic inflammation, which leads to insulin resistance [44]. A shift to M1 type has also been noted to contribute to liver inflammation and fibrosis in NAFLD: the M1/M2 polarization is an important factor in the progression of NAFLD. In the NASH model mice given β-cryptoxanthin, total macrophages were reduced, and the M1/M2 ratio was also decreased. At the same time, the expression of genes involved in M1/M2 polarization was suppressed in T cells (Figure 5). Thus, β-cryptoxanthin is expected to suppress oxidative stress and inflammatory responses as well as insulin resistance induced by chronic inflammation by reducing total macrophages and shifting their subsets to M2 dominance. As mentioned above, β-cryptoxanthin has been shown to contribute to the improvement of NAFLD through a multifaceted approach, including improved insulin resistance, suppression of oxidative stress and inflammation, and a reduction of macrophages and shift of their subsets (Figure 5) [43], which are also expected to have clinical applications.

## 5. Amelioration of NAFLD by Way of Activating PPAR γ through BCMO1-Mediated Retinoid Conversion of β-Cryptoxanthin

**Figure 6:**
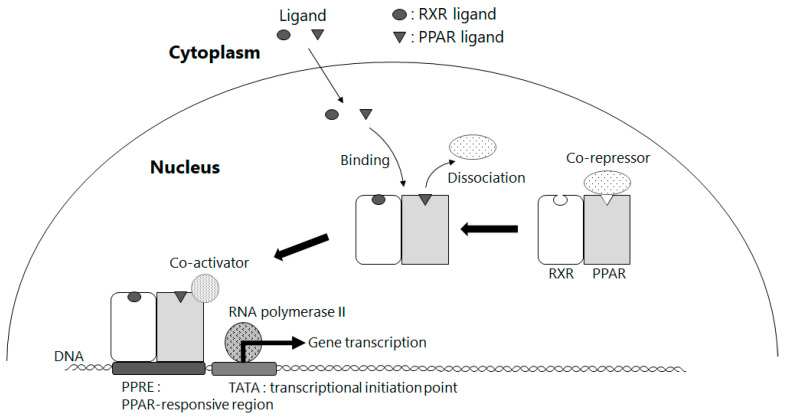
Expression mechanism of PPARs function by either RXR or PPAR ligands: RXR or PPAR ligands bind to and activate their corresponding receptors inside the nucleus, and then heterodimerized RXR/PPAR transcription factor binds to its responsive element in DNA and related gene transcription begins. RXR: retinoid X receptor, PPAR: peroxisome proliferator activated receptor, PPRE: peroxisome proliferator response element.

Next, we will discuss the relationship with peroxisome proliferator-activated receptors (PPARs) and β-cryptoxanthin. Since they are transcription factors in regulating genes involved in lipid and glucose metabolism, they are closely related to NAFLD, and many drug discovery studies have been conducted targeting PPARs [45]. There are three subtypes of PPARs (α, β, and γ), all of which form a heterodimer with RXR (PPAR-RXR) and regulate the expression of target genes in a ligand-dependent manner (Figure 6) [46,47]. PPARα is abundant in hepatic parenchymal cells and is mainly involved in the uptake and transport of fatty acids into cells and lipolysis, while PPARγ is primarily responsible for improving insulin sensitivity in adipocytes and is abundantly distributed in hepatic stellate cells of the liver. Typical exogenous ligands for PPARα and PPARγ are bezafibrate and pioglitazone, respectively, which are used as PPAR agonists to treat hyperlipidemia and insulin resistance, respectively. In particular, pioglitazone is a “first-hit” insulin resistance ameliorator for NAFLD, and pilot studies in NASH patients have reported its ameliorating effect on NASH [48,49]. PPARγ is highly expressed in hepatic stellate cells under normal liver conditions; however, its function is diminished in activated hepatic stellate cells along with their expression levels, while PPARγ ligands have also been reported to have an inhibitory effect on activated hepatic stellate cells [50]. These findings suggest that PPARγ activated by PPARγ ligand prevents hepatic fibrosis through inhibiting hepatic stellate cell activation and thus contributes to the pathological improvements for NASH.

The PPAR family is thought to be involved in the pathogenesis of NAFLD when its activity is regulated. As mentioned above, however, they are all PPAR-RXR heterodimers, and their activation requires stimulation by ligand binding to each of them. As shown in Section 3, NAFLD elicits abnormal retinoid metabolism and release of stored retinoids in the liver, suggesting that retinoic acid, a ligand for RXR, is deficient. RXR ligands are known to exhibit synergistic effects with PPAR ligands and single effects on RXR alone when activating heterodimers such as PPAR-RXR [51]. Therefore, in the pathosis of NAFLD, deficiencies in RXR ligands may cause abnormalities in the regulation of PPAR activity, which may further exacerbate the condition. BCMO1 knockout mice are reported to develop fatty liver due to disruption of lipid metabolism homeostasis as well as hypovitaminosis A (retinoid) status, and PPARγ expression is elevated in adipose tissue [52]. This suggests that retinoid production from provitamin A by BCMO1 plays an important role in regulating the activity of the PPAR family, which controls lipid metabolism. In a study on the organ distribution of β-cryptoxanthin in the body using cynomolgus monkeys as a model animal, β-cryptoxanthin ingested in the diet is confirmed to accumulate in high concentrations in the liver and adipose tissue [53]. BCMO1 was found in mice to be expressed not only in the liver and small intestine but also in adipose tissue [54]. These findings suggest that provitamin A and BCMO1, including β-cryptoxanthin, are involved in lipid metabolism along with the PPAR family. Deducing from these studies, β-cryptoxanthin, a provitamin A, may act as an RXR ligand by metabolic conversion to retinoids in vivo via BCMO1, contributing to the improvement of NAFLD pathology through a PPAR-mediated mechanism of action involved in improving insulin resistance.

## 6. Discussion

Many epidemiological and intervention studies have shown the usefulness of carotenoids in maintaining and improving human health. As the rapid increase in lifestyle-related diseases has become a major social problem, finding useful constituents in the foods we consume daily and applying them to preventive medicine will become increasingly important.

Carotenoids (α-carotene, β-carotene, and β-cryptoxanthin) belonging to the provitamin A family have been shown to be beneficial in preventing lifestyle-related diseases including obesity, type 2 diabetes, and liver dysfunction, especially through epidemiological studies in Japan. This review focuses on the effects of retinoids on NAFLD, which is rapidly increasing along with lifestyle-related diseases. Retinoids supplied by metabolic conversion from provitamin A may play an important role in NAFLD patients depleted in retinoids due to abnormal retinoid metabolism. This indicates that provitamin A maintained in high concentrations in vivo is of service for preventing NAFLD and the progression of the disease. Experiments using NASH-model mice also showed that β-cryptoxanthin prevented NAFLD by suppressing insulin resistance, lipid peroxidation due to oxidative stress, and inflammatory responses. Furthermore, β-cryptoxanthin was shown to shift the M1/M2 macrophage polarization toward M2 dominance, inducing anti-inflammatory and tissue repair.

This review also discussed the deep involvement of the PPAR family, a master regulator of lipid metabolism, in NAFLD, and the possibility that PPARs function as heterodimers with RXRs, and that in NAFLD and other liver disorders, retinoic acid deficiency due to abnormal retinoid metabolism could be one trigger, resulting in abnormal regulation of PPAR-RXR activity.

Provitamin A, β-cryptoxanthin in particular, has the potential to prevent lifestyle-related diseases from different angles, not only as an antioxidant but also as a retinoid precursor. Further research is expected to be developed on this possibility. BCMO1, which controls the metabolic conversion of provitamin A to retinoids, has polymorphic gene types in humans and is believed to be involved in the metabolic conversion described above [55]. It has been reported that the frequency of gene mutations that cause decreased BCMO1 activity is different among races, i.e., lower in Japanese and Chinese and higher in Europeans [56]. It will be intriguing to see whether there is an association between these polymorphic gene types of BCMO1 and the prevalence of NAFLD and other diseases caused by abnormalities in lipid metabolism. Further research is awaited.

We also look forward to large-scale clinical studies on the metabolism of provitamin A and the association between genetic polymorphisms of BCMO1 and the risk of developing NAFLD.

## 7. Conclusions

Of six dietary carotenoids, α-carotene, β-carotene, and β-cryptoxanthin, called provitamin A, are metabolically converted to vitamin A in the body. Lifestyle-related diseases are increasing and have been a serious issue in modern society. Carotenoids and carotenoid-rich green and yellow vegetables have been reported to reduce the risk of development of lifestyle-related diseases. β-Cryptoxanthin has recently gained attention for its risk-reducing effects especially on non-alcoholic fatty liver disease (NAFLD), from epidemiological, interventional, and mechanistic studies.

Epidemiological studies indicate that oral intake of β-cryptoxanthin is effective not only in reducing the risk of developing NAFLD but also in improving the post-disease conditions and that the reduction of oxidative stress is considered one of its mechanisms. Additionally, retinoids have been reported to be useful as a therapeutic agent for NAFLD, but their overconsumption can cause adverse effects, while provitamin A molecules such as β-cryptoxanthin do not cause adverse effects and are known to serve as a supply source of retinoids through metabolic conversion by BCMO1 only when retinoids are deficient in the body. Elimination of stored retinoids due to abnormal retinoid metabolism or activation of hepatic stellate cells results in retinoid deficiency, where metabolic conversion or consumption of provitamin A is expected to be enhanced. This mechanism may be responsible for the inverse correlation between the incidence and progression of NAFLD and serum provitamin A concentration, as observed in the epidemiological studies.

From mechanistic studies using NAFLD-model mice, β-cryptoxanthin has been shown to contribute to the improvement of NAFLD through a multifaceted approach, including improved insulin resistance, suppression of oxidative stress and inflammation, and a reduction of macrophages and a shift of their subsets, which are also expected to have clinical applications. Additionally, β-cryptoxanthin and BCMO1 are suggested to be involved in lipid metabolism along with the PPAR family. β-Cryptoxanthin may act as an RXR ligand by metabolic conversion to retinoids in vivo via BCMO1, contributing to the improvement of NAFLD pathology through a PPAR-mediated mechanism of action involved in improving insulin resistance.

β-Cryptoxanthin has the potential to prevent lifestyle-related diseases from different angles, not only as an antioxidant but also as a retinoid precursor. BCMO1, which controls the metabolic conversion of provitamin A to retinoids, has polymorphic gene types in humans and is believed to be involved in the metabolic processes described above. It will be intriguing to investigate whether there is an association between these polymorphic gene types of BCMO1 and the prevalence of NAFLD and other diseases caused by abnormalities in lipid metabolism.

## Figures and Tables

**Table 1:** Assessment of relationships between the carotenoid contents in blood or carotenoid consumption and risk of lifestyle-related diseases from several cohort studies.

	Disorder of Lipid Metabolism	Type 2 Diabetes		Non-Alcoholic Fatty Liver Disease		
Test areas	Japan [20] (Mikkabi)	Japan [21] (Mikkabi)	Finland [22] (Several areas)	Japan [23] (Mikkabi)	China [24] (Guangzhou)	Japan [25] (Ehime Univ. Hospital)
Subjects	General inhabitants	General inhabitants	General inhabitants	General inhabitants	General inhabitants	Patients
**Provitamin A**						
α-carotene	Excellent	Excellent	None	Fair	Excellent	None
β-carotene	Excellent	None	None	Excellent	Excellent	None
β-cryptoxanthin	Excellent	Excellent	Excellent	Excellent	Excellent	Excellent
**Non-provitamin A**						
Lycopene	None	None	None	None	Excellent	None
Lutein	None	None	None	None	Excellent	None
Zeaxanthin	None	None	None	None	Excellent	None

Excellent: There are significantly negative correlations between the carotenoids and risk of diseases. Fair: There are not significantly negative correlations but negative tendencies between the carotenoids and risk of diseases. None: There are neither significantly negative correlations nor tendencies between the carotenoids and risk of diseases.

## Abbreviations

ADH	alcohol dehydrogenase
AKR1B10	aldo-keto reductase family 1 member B10
ALDH1A	retinaldehyde dehydrogenase 1 family, member A
ALT	alanine aminotransferase
AST	aspartate amino group transferase
BCMO1	β-carotene 15,15′-monooxygenase 1
BMI	body mass index
CRABP	cellular retinoic acid-binding protein
CRBP	cellular retinol-binding protein
CYP	cytochrome P450 family members
DGAT	diacylglycerol acyltransferase
DHRS	membrane-bound short-chain dehydrogenases/reductases
γ-GTP	γ-glutamyl transpeptidase
IL-1	interleukin 1
IL-6	interleukin 6
LDL	low density lipoprotein
LRAT	lecithin retinol acyltransferase
MS	metabolic syndrome
NAFL	non-alcoholic fatty liver
NAFLD	non-alcoholic fatty liver disease
NASH	non-alcoholic steatohepatitis
PPAR	peroxisome proliferator-activated receptor
PPRE	peroxisome proliferator response element
RAR	retinoic acid receptor
RBP4	retinol-binding protein 4
RDH	retinal dehydrogenase
REH	retinyl ester hydrolase
RXR	retinoid X receptor
SOAC	singlet oxygen absorption capacity
SRB-1	scavenger receptor class B type 1
TATA	transcriptional initiation point
TNF-α	tumor necrosis factor-α
UV	ultraviolet light
